# Bacterial community structure and novel species of magnetotactic bacteria in sediments from a seamount in the Mariana volcanic arc

**DOI:** 10.1038/s41598-017-17445-4

**Published:** 2017-12-21

**Authors:** Jia Liu, Wenyan Zhang, Xuegong Li, Xuegang Li, Xumiao Chen, Jin-Hua Li, Zhaojie Teng, Cong Xu, Claire-Lise Santini, Li Zhao, Yuan Zhao, Heng Zhang, Wei-Jia Zhang, Kuidong Xu, Chaolun Li, Yongxin Pan, Tian Xiao, Hongmiao Pan, Long-Fei Wu

**Affiliations:** 1 CAS Key Laboratory of Marine Ecology and Environmental Sciences, Institute of Oceanology, Chinese Academy of Sciences, Qingdao, 266071 China; 2 Laboratory for Marine Ecology and Environmental Science, Qingdao National Laboratory for Marine Science and Technology, Qingdao, 266071 China; 3 University of Chinese Academy of Sciences, Beijing, 100864 China; 4 Laboratory of Deep Sea Microbial Cell Biology, Institute of Deep Sea Science and Engineering, Chinese Academy of Sciences, Sanya, 572000 China; 5 International Associated Laboratory of Evolution and Development of Magnetotactic Multicellular Organisms (LIA-MagMC), CNRS-CAS, Marseille-Beijing-Qingdao-Sanya, Qingdao, 266071 China; 6 Department of Marine Organism Taxonomy and Phylogeny, Institute of Oceanology, Chinese Academy of Sciences, Qingdao, 266071 China; 7 Paleomagnetism and Geochronology Lab, Key Laboratory of the Earth’s Deep Interior, Institute of Geology and Geophysics, Chinese Academy of Sciences, Beijing, 100029 China; 8 Aix Marseille University, CNRS, LCB, Marseille, France

## Abstract

Seamounts are undersea mountains rising abruptly from the sea floor and interacting dynamically with underwater currents. They represent unique biological habitats with various microbial community structures. Certain seamount bacteria form conspicuous extracellular iron oxide structures, including encrusted stalks, flattened bifurcating tubes, and filamentous sheaths. To extend our knowledge of seamount ecosystems, we performed an integrated study on population structure and the occurrence of magnetotactic bacteria (MTB) that synthesize intracellular iron oxide nanocrystals in sediments of a seamount in the Mariana volcanic arc. We found Proteobacteria dominant at 13 of 14 stations, but ranked second in abundance to members of the phylum Firmicutes at the deep-water station located on a steep slope facing the Mariana-Yap Trench. Live MTB dwell in biogenic sediments from all 14 stations ranging in depth from 238 to 2,023 m. Some magnetotactic cocci possess the most complex flagellar apparatus yet reported; 19 flagella are arranged in a 3:4:5:4:3 array within a flagellar bundle. Phylogenetic analysis of 16S rRNA gene sequences identified 16 novel species of MTB specific to this seamount. Together the results obtained indicate that geographic properties of the seamount stations are important in shaping the bacterial community structure and the MTB composition.

## Introduction

Seamounts are unique oceanic ecosystems. They extend from the ocean floor but remain below the water surface, occur at various depths in all oceans worldwide, and vary in form and size^1^. Because of their physical and geographical features, seamounts affect underwater currents and create upwellings that attract plankton, corals, fish, and marine mammals, establishing biological hotspots in the ocean^1–3^. It has been hypothesized that seamounts support high levels of biodiversity and endemism of marine animals^4^. Characterization of microbial populations in sediments has been undertaken for the Marsili and Palinuro seamounts in the Tyrrhenian Sea^5–8^; the Takuyo-Daigo seamount in the northwest Pacific Ocean^9^; the Suiyo seamount in the western Pacific Ocean^10^; and the Afanasy Nikitin seamount, which is located in the equatorial East Indian Ocean^11^. Bacterial abundance and biomass were significantly higher in sediments of the Marsili and Palinuro seamounts than in non-seamount sediments^5^. Gammaproteobacteria dominated the microbial communities in both the seamount and non-seamount sediments, and microdiversity of Bacillales and Actinobacteria was found^6–8^. Other studies have shown that Gammaproteobacteria were abundant in sediments surrounding ferromanganese crusts at the Takuyo-Daigo seamount, while Alphaproteobacteria and Gammaproteobacteria were dominant in sand samples from a hydrothermal field at the Suiyo seamount^9,10^. Moreover, at 150 cm depth sediments collected from Afanasy Nikitin seamount were dominated by clones belonging to the Firmicutes and Gammaproteobacteria, while Gammaproteobacteria and Betaproteobacteria were dominant in sediments at 200 cm depth^11^. In this study, we performed a systematic investigation of the bacterial diversity and community structure in sediments collected from 14 stations on a seamount located in the tropical western Pacific Ocean; additionally, this cruise marks the first time this seamount has been surveyed. We focused on the occurrence of seamount magnetotactic bacteria (MTB) that are widely distributed in freshwater and marine ecosystems^12–16^. MTB are a morphologically, phylogenetically, and physiologically diverse group of bacteria that produce single-domain magnetite (Fe_3_O_4_) or greigite (Fe_3_S_4_) crystals in intracellular organelles called magnetosomes. The biogenesis of magnetosomes is a genetically controlled process^17^. Magnetosomes arranged in a chain result in a magnetic dipole moment of cells, which enables them to align and swim along geomagnetic field lines and migrate to the oxic-anoxic interface in chemically stratified sediments of freshwater or marine environments. Phylogenetic analysis based on the 16S rRNA gene sequences affiliates the MTB to Proteobacteria, Nitrospirae, Omnitrophica and the candidate phylum Latescibacteria^18–20^. Although they are ubiquitous in coastal marine sediments, MTB have only rarely been reported in deep-sea habitats, mainly because of the technical difficulties inherent in accessing these environments^21–28^. MTB were first collected from hemipelagic sediments in the Santa Barbara basin (34°14′N, 120°1′W) at a depth of 598 m^25^. The magnetic crystals of magnetosomes are thought to be a significant source of magnetic remanence in marine sediments, and their presence has been used as fossil evidence for the occurrence of MTB in marine sediments at water depths ranging from approximately 600 m to 3,000 m^25,29,30^. Observations of live MTB in pelagic and hemipelagic sediments at depths up to approximately 3,000 m in the eastern South Atlantic Ocean have been reported^31^. Metagenomic and restriction fragment length polymorphism approaches applied to deep-sea (>5,000 m) sediments from the polymetallic nodule province in the east and northeast equatorial Pacific Ocean have identified 16S rRNA gene sequences closely related to *Magnetospirillum* species and *Magnetovibrio blakemorei* strain MV-1, respectively^32,33^. Recently, based on phylogenetic and magnetite analyses, we reported fossil evidence for the occurrence of MTB in deep-sea sediments from the eastern Pacific manganese nodule province, at water depths of 4,970–5,620 m^13^. Live MTB have only been observed at one site on one seamount at depth of 1,007 m^31^. We still do not know how widely MTB are distributed in this unique ecosystem.

In this study, we investigated microbial communities and occurrence of MTB on a seamount of the Mariana volcanic arc near the Challenger Deep in the tropical western Pacific Ocean. We observed distinct population structures in the bacterial communities that were dependent on the geographic properties of the seamount, and we detected MTB belonging to novel species, including some with unique flagella architecture. These findings extend our knowledge of the microbial communities present in seamount ecosystems.

## Results

### Sampling station descriptions and chemical characterization of sediments

**Figure 1:**
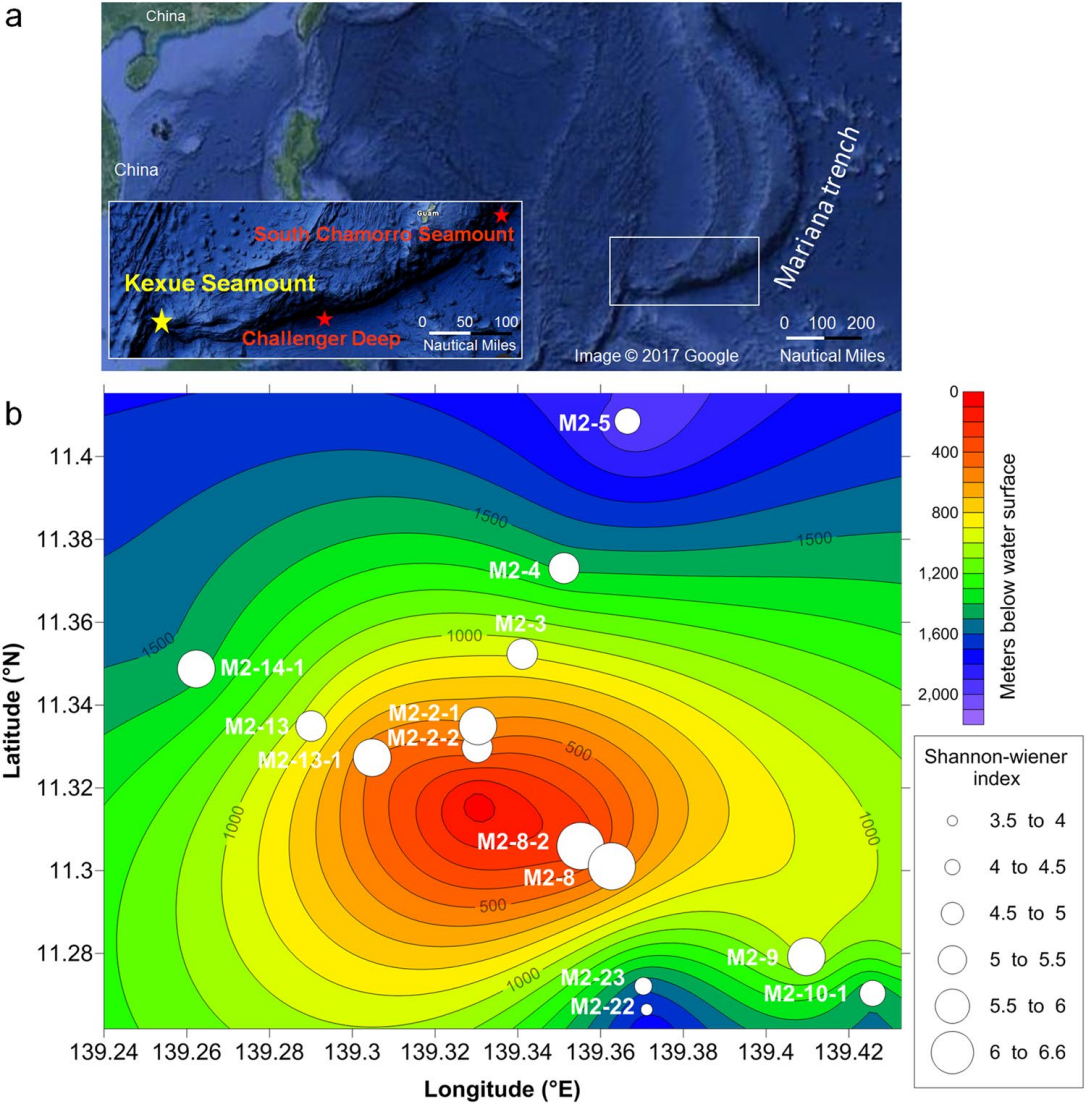
Sampling stations and bacterial diversity at each station. Panel (**a**) Location of the Kexue seamount (yellow stars) is shown in the insert. The South Chamorro seamount and Challenger Deep are indicated with red stars. The map data was from Imagery ©2017 Data SIO, NOAA, U.S. Navy, NGA, GEBCO, Map data ©2017 Google, ZENRIN downloaded at https://www.google.fr/maps/@11.7463718,145.5037996,1320412 m/data = !3m1!1e3. The fourteen ROV sampling stations at the Kexue seamount are indicated with white circles and their unique station code. Panel (**b**) The diameters of the white circles are proportional to the Shannon-Wiener index for the bacteria community structure in sediments.

The sampling stations are located on a previously unreported seamount, referred to as the Kexue seamount, in a zone from 139.1417°E to 139.5518°E and 11.1586°N to 11.4818°N in the Mariana volcanic arc. The Kexue seamount was discovered during this cruise by using Multibeam SeaBeam 3012 (Wärtsilä ELAC, Kiel, Germany). This area is located approximately 740 km southwest of the South Chamorro seamount and 340 km west of the Challenger Deep (Fig. 1a). The peak of the seamount rises to approximately 20 m below the water surface. The 14 sampling stations, located at depths ranging from 238 m to 2,023 m, were selected based on remotely operated vehicle (ROV) operations and safety. Viewed from the top of this seamount, three stations (M2-13-1, M2-13, and M2-14-1) were distributed in the three-figure-bearings direction of 300°, 5 stations (M2-2-1, M2-2-2, M2-3, M2-4, and M2-5) were distributed similarly at a bearing of 030°, and another 4 stations (M2-8, M2-8-2, M2-9, and M2-10-1) were distributed at a bearing of 130° (Fig. 1b). Most stations were on gentle slopes of the seamount, but we also collected samples from two stations (M2-22 and M2-23) on the steep southeast slope (approximately 150°), which faces the direction of the Mariana Trench (Fig. 1).

Stereomicroscope examination of the Kexue seamount sediment composition showed that sediments were mainly composed of various proportions of coral and foraminiferan sands. Brownish to white-yellowish crust-like coral sands interspersed with sparse foraminifera (Supplementary Fig. S1; stations M2-9, M2-2-2, M2-2-1, M2-8, and M2-8-2). Other sediments were composed primarily of foraminiferan sands (M2-3, M2-10-1, and M2-22) or were interspersed with sparse coral sands (M2-5), crusted coral particles (M2-14-1), partially disintegrated foraminiferan tests (M2-23), or fine yellowish particles (M2-4). The sediments from stations M2-13 and M2-13-1 were fine, white-coral sands, with sparse amounts of foraminiferan sands.

**Figure 2:**
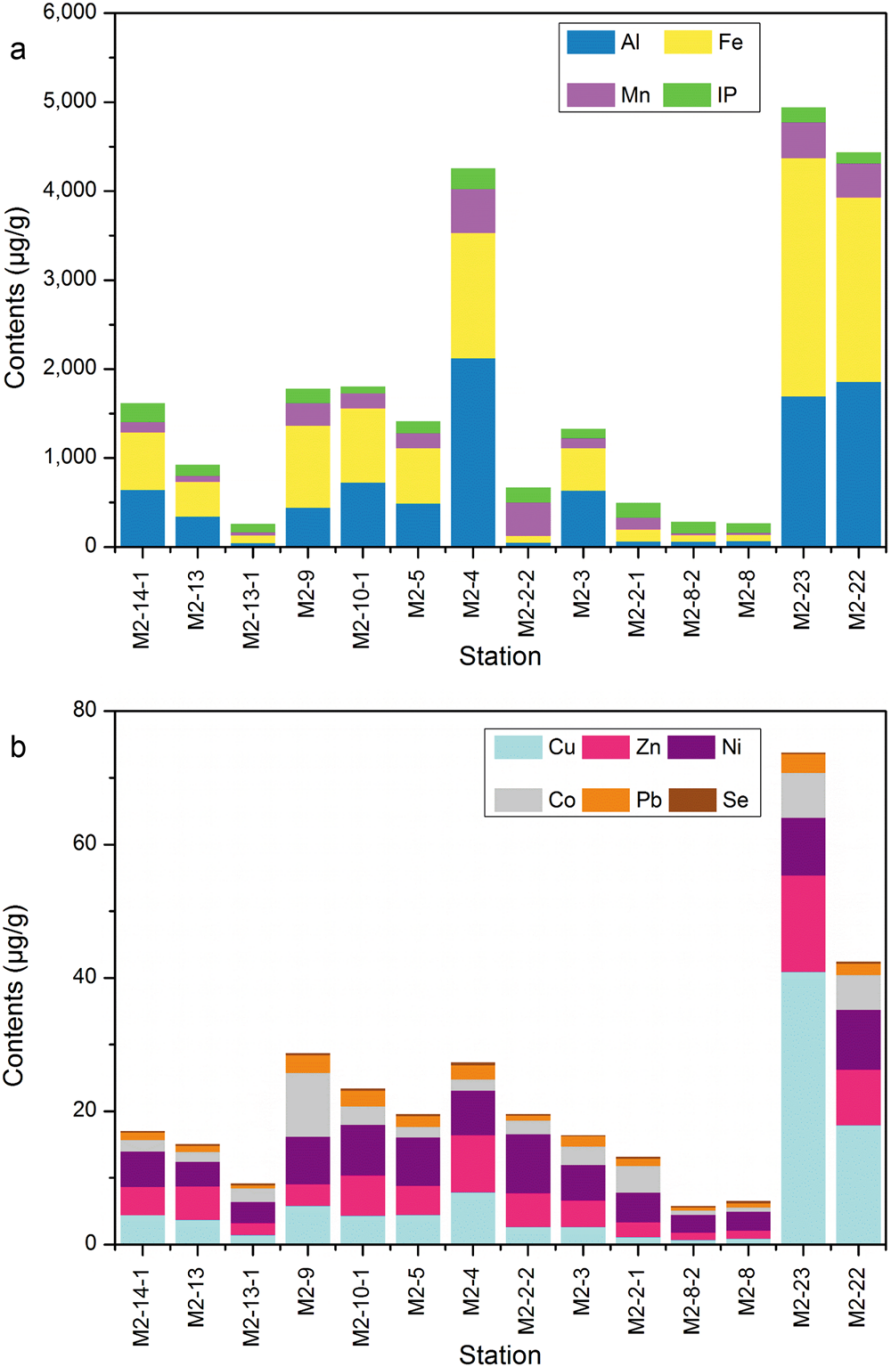
The concentrations of chemical elements in the sediments of the Kexue seamount. Panels a and b show the concentrations (μg/g) of major and trace elements, respectively, at the 14 stations.

Geochemical analysis showed that, at shallow stations within 600 m below the water surface (Fig. 1b; stations M2-8, M2-8-2, M2-2-1, M2-2-2 and M2-13-1), the concentrations of major elements were low (Fig. 2a). The dominant major elements were inorganic phosphorus, which was found at stations M2-8, M2-8-2, M2-2-1, and M2-13-1 (Fig. 2a; green bars), and Mn, which was found at station M2-2-2 (Fig. 2a; pink bar). Stations M2-22, M2-23, and M2-4 were distinguished from the other stations because they had 3- to 40-fold higher concentrations (1.9 × 10^3^, 1.7 × 10^3^, and 2.1 × 10^3^ μg/g, respectively) of Al (Fig. 2a; blue bars) and 3- to 37-fold higher concentrations (2.1 × 10^3^, 2.7 × 10^3^, and 1.4 × 10^3^ μg/g, respectively) of Fe (Fig. 2a; yellow bars) than the other stations. Interestingly, sediments from the stations M2-22 and M2-23 had markedly higher concentrations of trace elements (Fig. 2b), particularly Cu (Fig. 2b; light blue bars), for which the concentration was 5- to 60-fold higher than the other stations.

### Microbial population in sediments of the 14 seamount sampling stations

The microbial communities in the sediments from the seamount sampling stations were investigated by analysing the bacterial (16S rRNA gene) community structure using amplicon sequencing. A total of 443,433 valid reads and 2,269 operational taxonomic units (OTUs) (based on 97% sequence similarity) were obtained from the 14 stations. The populations were well represented because the rarefaction curves indicated that sufficient sequencing depths were achieved (Supplementary Fig. S2). The Good’s coverage estimates for all samples exceeded 0.99 (Supplementary Table S1). The Shannon-Wiener index showed that the highest community diversity occurred on the top of the seamount, and the community complexity decreased significantly with the increasing depths of the sampling stations (Fig. 1b). Pearson correlation analysis between the microbial diversity and the chemical elements in the sediments showed that the Shannon-Wiener index was highly negatively correlated with the concentrations of Al, Fe, Cu, Zn, and Ni (P value: 0.005, 0.001, 0.003, <0.001 and 0.001, respectively) and negatively correlated with the concentrations of Mn and Pb (P value: 0.013 and 0.017, respectively) (Supplementary Table S2). The least complex community was at station M2-22 (depth of 1,676 m) on the steep slope facing the Mariana Trench (Fig. 1b; Supplementary Table S1).

**Figure 3:**
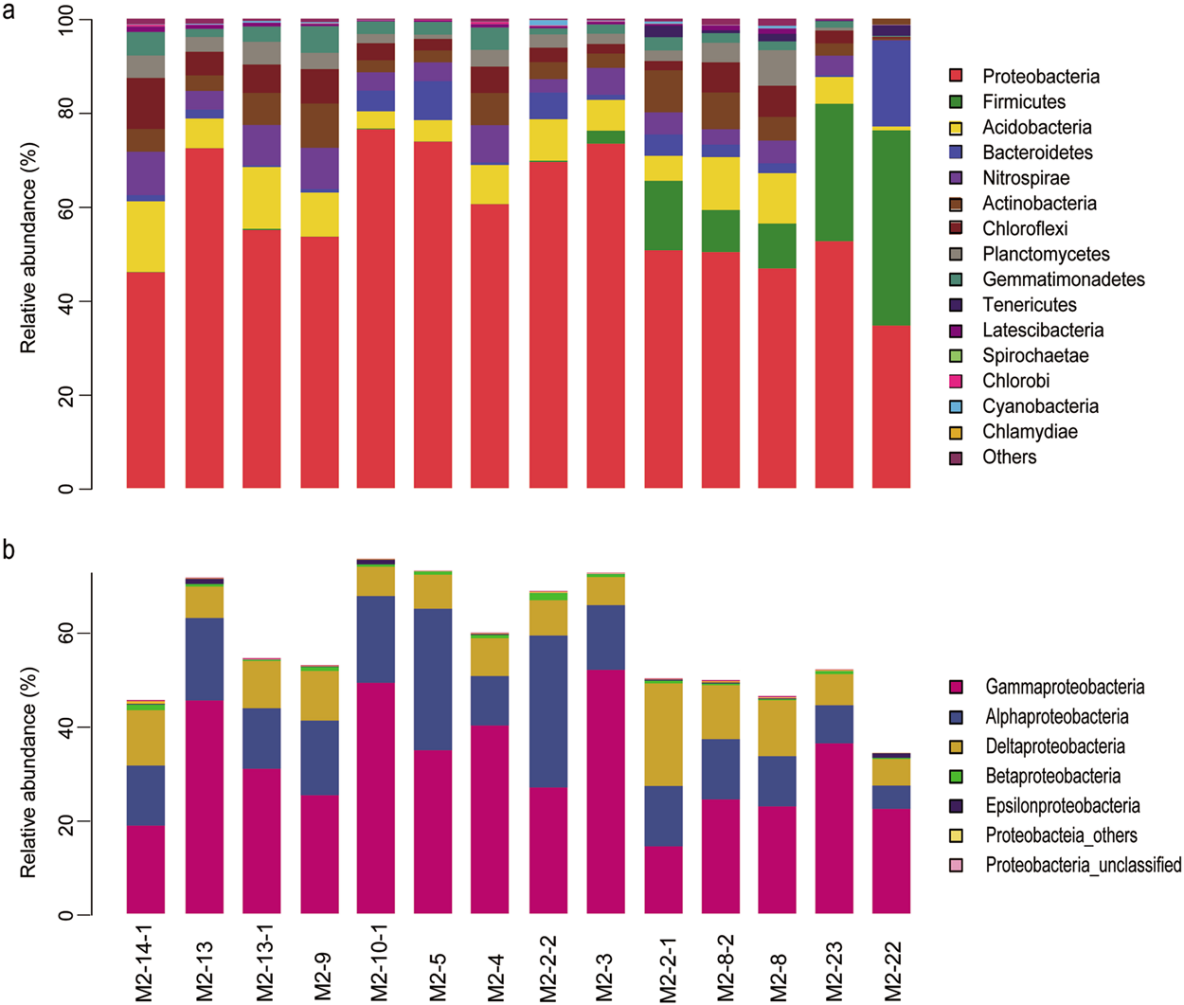
Bacterial 16 rRNA gene community composition at 14 stations at the Kexue seamount. Panel (**a**) Taxonomic assignments were obtained from Illumina MiSeq sequencing datasets, using the RDP classifier tool and the Silva database, respectively. Only phyla with a relative abundance ≥1% are shown. Phyla with a relative abundance <1% were grouped as ‘Other’. Panel (**b**) Taxonomic assignments at the class level within the phylum Proteobacteria.

The taxa analysed in the communities ranged from genus to phylum levels. The phylum Proteobacteria (Fig. 3a; red bars) dominated the communities at all sampling stations except M2-22, where the phylum Firmicutes (Fig. 3a; green bars) was the most abundant (41.6% compared with 34.6% for Proteobacteria). Within the phylum Proteobacteria, Gammaproteobacteria was the most abundant class at most stations (the proportion ranged from 22.6% to 52.5%), while Alphaproteobacteria and Deltaproteobacteria were the most important classes at M2-2-2 and M2-2-1, accounting for 32.7% and 22.1%, respectively (Fig. 3b). Interestingly, Firmicutes were relatively abundant at stations M2-22, M2-23, M2-8, M2-8-2, M2-2-1 and M2-3, which were aligned along the steep southeast slope (150°) from depth to the top extending toward to the 330° direction (Figs 3a and 1b). The abundance of Firmicutes at these stations decreased in the order from the depth to the top. Notably, at station M2-22 approximately 18.3% of the relative community abundance comprised members of the phylum Bacterioidetes (Fig. 3a; blue bars), which was 2- to 40-fold higher than at the other stations. The fourth most abundant bacterial population at station M2-22 belonged to the phylum Tenericutes (Fig. 3a; dark blue bars); this population was only detected at stations on the steep southeast slope (i.e., M2-22, M2-23, M2-8, M2-8-2, and M2-2-1). The fifth most abundant bacterial population at station M2-22 belonged to the phylum Acidobacteria (Fig. 3a; yellow bars), and the abundance of this phylum was 4- to 18-fold lower than at the other stations. All other phyla, including Nitrospirae (Fig. 3a; violet bars), Actinobacteria (brown bars), Chloroflexi (dark red bars), and Planctomycetes (grey bars), accounted for <0.4% of the community abundance.

### Magnetotactic bacteria in Kexue seamount sediments

According to BLAST results based on the NCBI nr/nt database, 21 OTUs, with a total of 1,130-sequence reads from amplicon sequencing, were related to MTB. MTB sequences were detected at all sampling stations except M2-22 (Table 1). Two other deep stations, M2-23 and M2-5, had the lowest number of MTB reads (13 reads each) (Table 1). In contrast, stations M2-8 and M2-8-2, which are both near the top of the seamount, had relatively high MTB diversity (184 and 121 reads, respectively) (Table 1). Based on the RDP classifier tool, taxonomic classification of each MTB OTU indicated that all OTUs belonged to Alphaproteobacteria and were affiliated with the genera *Magnetospira* and *Magnetovibrio* (Supplementary Fig. S3). The OTU2037 (MF073302) (216 reads) belonged to the genus *Magnetospira*; this OTU was the most abundant one associated with the Kexue seamount, and, together with OTU1252 (MF073294), was the most widely distributed. These two OTUs were detected at all sampling stations except M2-22 and M2-5, which are the two deepest of our sampling stations on the seamount. The MTB were more abundant at shallow depths, rather than deeper depths. With respect to geochemical features, the number of MTB reads was negatively correlated with the sediment Ni content, and the number of MTB OTUs was negatively correlated with the sediment Fe, Mn, Cu, and Ni contents (Supplementary Table S2).

**Table 1:** MTB abundance and MTB reads at 14 stations at the Kexue seamount.

**Station**	**Longitude (139°E)**	**Latitude (11°N)**	**Depth (m)**	**Total reads**	**MTB reads**	**Percentage of MTB reads (%)**	**MTB OTUs**	**MTB abundance (inds.dm** ^**−3**^ **)**
M2-14-1	15′44″	20′55″	1, 427	26,252	128	0.49	13	70
M2-13	17′24″	20′06″	939	35,695	204	0.57	12	49
M2-13-1	18′17″	19′38″	491	27,072	36	0.13	9	9
M2-9	24′35″	16′45″	980	26,739	98	0.37	10	226
M2-10-1	25′33″	16′13″	1, 493	37,520	73	0.19	12	7
M2-5	21′59″	24′31″	2, 023	32,069	13	0.04	3	49
M2-4	21′04″	22′23″	1, 420	35,320	100	0.28	10	15
M2-2-2	19′49″	19′47″	475	35,092	85	0.24	9	107
M2-3	20′28″	21′08″	911	39,274	51	0.13	9	120
M2-2-1	19′49″	20′06″	575	26,252	24	0.09	9	33
M2-8-2	21′19″	18′21″	238	28,749	121	0.42	14	18
M2-8	21′46″	18′04″	495	25,797	184	0.71	14	9
M2-23	22′13″	16′19″	1, 583	32,791	13	0.04	5	10
M2-22	22′16″	15′59″	1, 676	30,474	0	0.00	0	43

Magnetotactic bacteria from the Kexue seamount were identified using two methods. MTB reads were obtained by metagenomics analysis. MTB abundance (inds./dm^3^) was based on the number of live MTB directly observed by microscopy and adjusted to account for the volume of sediment from which the counts was made.

**Figure 4:**
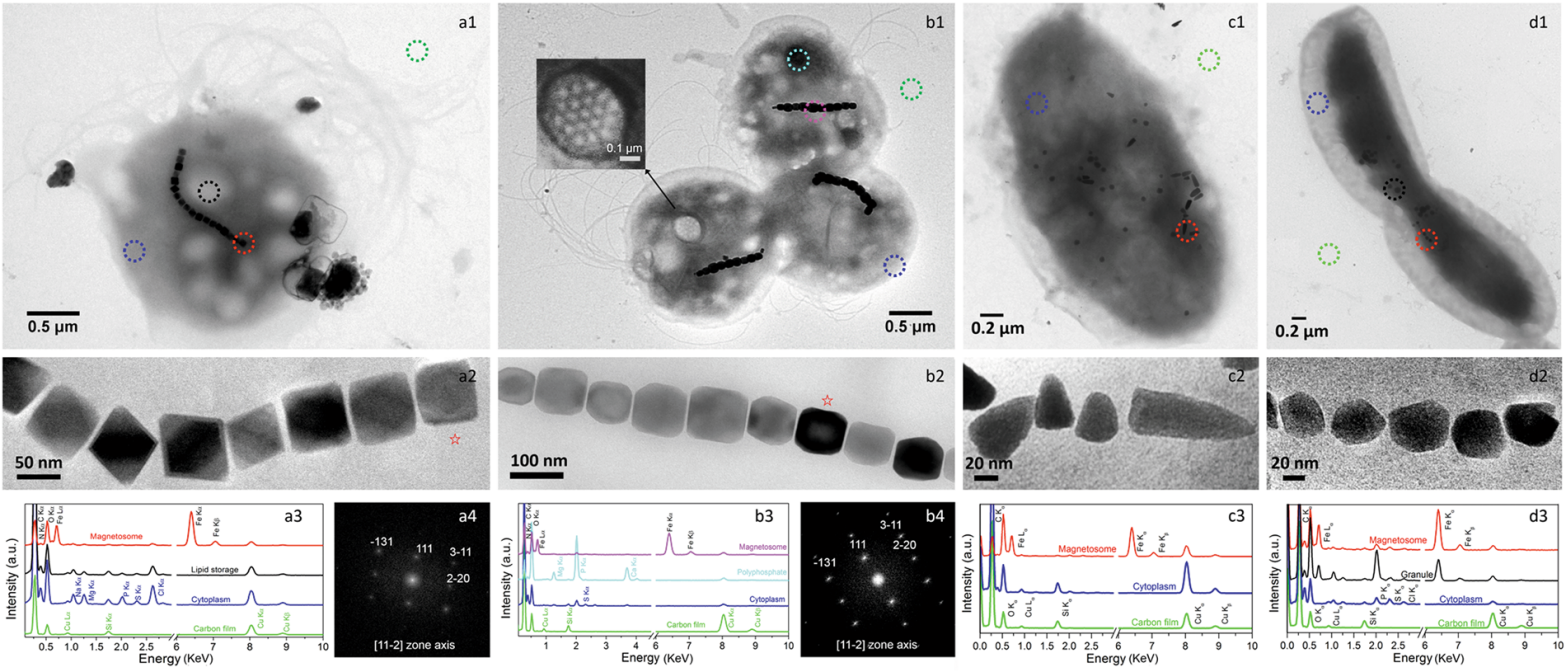
Seamount MTB and identification of the magnetic crystals. Panels a1 and b1: Magnetotactic cocci having single chains of octahedral and prismatic magnetite magnetosomes. Panel c1 and d1: Vibrioid MTB and dumbbell-shaped MTB. Panels a2, b2, c2 and d2: Magnified images of magnetosomes. Coloured circles indicate analysis points for the EDXS analyses shown in a3, b3, c3 and d3. Electron diffraction images of the red star magnetosomes are shown in a4 and b4. The inset in b1 shows a flagella base platform comprising 19 individual flagella that are arranged in a 3:4:5:4:3 array.

The metagenomics analysis provided global information about the potential occurrence of MTB at the stations. By using the observation of magnetotactic behaviour as a criterion we found that MTB were present at all stations with depths between 238–2,023 m (Table 1). Station M2-10-1, at a depth of 1,493 m, had the minimum abundance of live MTB (7 individuals dm^-3^), and the maximum abundance of live MTB was found at station M2-9 (depth of 980 m, 226 individuals dm^-3^) (Table 1). Transmission electron microscopy (TEM) indicated the presence of four morphotypes of MTB in the Kexue seamount sediments (Fig. 4). Magnetotactic cocci dominated (95.4%) (Fig. 4a1 and b1); these contained various types of magnetosomes, including octahedral (Fig. 4a2) and prismatic forms (Fig. 4b2). In addition, we observed vibrioid MTB (0.3%) with bullet-shaped magnetosomes (Fig. 4c1 and c2) and dumbbell-shaped MTB (4.3%) with irregular-shaped magnetosomes (Fig. 4d1 and d2). Energy dispersive X-ray spectroscopy (EDXS) analysis revealed significantly higher levels of O and Fe associated with the magnetosomes than in the areas of the cytoplasm without magnetosomes, indicating that the magnetosomes were composed of iron oxide (Fig. 4a3 b3, c3 and d3). High-resolution TEM and electron diffraction analysis showed that the magnetosomes were structurally related to magnetite (Fig. 4a4 and b4). TEM revealed that magnetotactic coccoid cells had one or two flagellar bundles, each comprising 19 individual flagella with diameters of 17.75 ± 2.79 nm (n = 27). The 19 flagella were organized in a 3:4:5:4:3 array (Fig. 4b1 inset and Supplementary Fig. S4). It represents a new type of highly organized and unique motility apparatus.

### Phylogenetic relationships of seamount MTB

**Figure 5:**
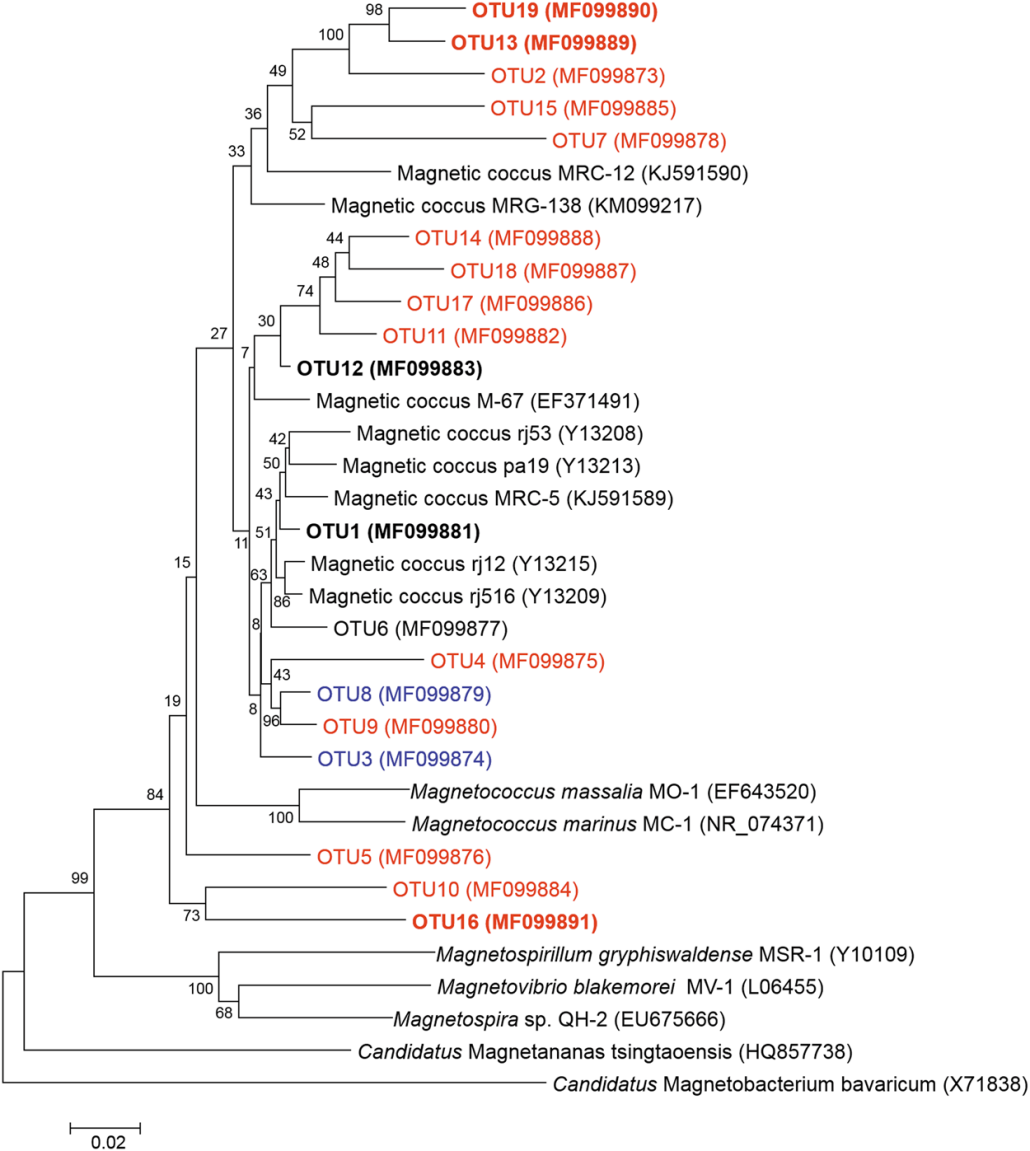
Phylogenetic tree based on 16S rRNA gene sequences for the 19 MTB OTUs identified from live MTB samples. The phylogenetic tree was constructed based on neighbor-joining analysis. Bootstrap values at the nodes are percentages of 1,000 replicates. We used 97% and 95% sequence identity as the thresholds for classifying to species and genus levels, respectively. OTUs belonging to new species are indicated in blue. OTUs belonging to new genera are indicated in red. OTUs identified from micromanipulated samples are emphasized in bold characters. The scale bar indicates 2% sequence divergence.

During laboratory storage, the abundance of MTB increased by up to four orders of magnitude in the sediments from the shallowest station (M2-8-2) and in two suction-collected sediment samples; this made it possible to amplify, clone, and sequence the 16S rRNA genes. Based on a 97% sequence identity threshold, with almost the full length of the 16S rRNA gene, we obtained a total of 19 OTUs related to the reported MTB (Fig. 5, Supplementary Table S3). Among the 19 OTUs, 5 OTUs (OTU11/12/14/17/18) showed a >97% sequence identity with OTU302, based on amplicon sequencing of laboratory-stored samples. All MTB OTUs from the Kexue seamount were affiliated with Alphaproteobacteria (Fig. 5). When queried against the NCBI nr/nt database, the highest level of sequence identity was with magnetotactic cocci collected from Huiquan Bay and Yuehu Lagoon (Yellow Sea) in the West Pacific Ocean and magnetotactic bacteria from Itaipu Lagoon (Rio de Janeiro, Brazil) in the West Atlantic Ocean (Supplementary Table S3). We use 97% and 95% sequence identity as the thresholds for classifying species and genera, respectively^34^. The 19 OTUs included 16 new species belonging to 12 novel genera (encompassing 14 of the new species) and one known genus (2 species) (Fig. 5, Supplementary Table S3). Therefore, the 12 new genera may represent MTB endemic to the Kexue seamount.

## Discussion

The geochemical and hydrological conditions of seamounts play very important roles in determining the microbial biodiversity that occurs in these unique ecosystems^35,36^. Populations of Gammaproteobacteria are dominant in the sediments of the Marsili and Palinuro seamounts^6^ and the Takuyo-Daigo seamount^9,10^. Interestingly, Firmicutes and Gammaproteobacteria were dominant in the upper sediments from the Afanasy Nikitin seamount, but Gammaproteobacteria and Betaproteobacteria were dominant in sediments at 200 cm depth^11^. Epsilonproteobacteria and Zetaproteobacteria have been reported to dominate the microbial community of the South Chamorro seamount^35^, which is 740 km away from the Kexue seamount. Our study showed that Gammaproteobacteria were dominant in the sediments from most stations at the Kexue seamount; the only exception was the deep station (M2-22) on the steep slope that faces the Mariana-Yap Trench, where Firmicutes were the most common members. This station is distinguishable from the other stations in terms of geochemical composition (Fig. 2), community structure (Fig. 3), and the occurrence of magnetotactic bacteria (Table 1). Differences between the microbial communities of the South Chamorro and the Kexue seamounts may be a result of their distinct geological characteristics. For example, the South Chamorro seamount is an alkaline (up to pH 12.5), serpentinite mud volcano with benthic megafauna, including mussels, gastropods, tubeworms, and galatheid crabs^37^. In contrast, the Kexue seamount is rich in sponges and corals, and the sediments mainly consist of coral and foraminiferan sands (Supplementary Fig. S1). The different community compositions may be a consequence of the heterogeneous sediment and the complex geochemistry of these seamounts.

In terms of metabolic types, seamounts are dominated by iron-oxidizing (FeOB), hydrogen- or sulfur-oxidizing (HSOB), sulfur-oxidizing (SOB), sulfur-reducing (SRB), and anaerobic methane-oxidizing (AMOB) microbes^35^. Certain bacteria at these seamounts form conspicuous extracellular iron oxide structures, including encrusted stalks, flattened bifurcating tubes, and filamentous sheaths^2^. MTB with accumulated intracellular iron oxide minerals have been observed at only one station at the Walvis Ridge seamount in the South Atlantic Ocean^31^. In this study, we found live MTB in sediments from all 14 stations at the Kexue seamount. They were composed of 19 OTUs, including 12 new genera that may represent MTB endemic to the Kexue seamount. Interestingly, to date, MTB have only been reported in sediments composed of finely divided rock and mineral particles, (Supplementary Fig.S1, panel o). In contrast, the Kexue seamount sediments are of biogenic origin, making this the first report of MTB occurring in biogenic sediments.

Metagenomic analysis showed that *Magnetospira* and *Magnetovibrio* are the most abundant MTB but microscope observation revealed that magnetotactic cocci and vibrio are the most dominant morphotypes. This discrepancy might be due to the analysis method used. In most cases, metagenomic analysis reflects dominant populations; however, the magnetic collection used for microscope observations is more efficient at collecting magnetic cocci. In addition, the molecular markers detected at a given station may have originated from bacteria living in other places that were brought to the sampling stations. The best evidence for the presence of MTB in the sediments from sampling sites is the direct observation of live magnetotactic bacteria in the samples.

Seamounts rise abruptly from the ocean seafloor and have a substantial impact on physical oceanography: they determine the nature of circulation, local hydrographic distributions, turbulence, and vertical and horizontal nutrient transport^36^. It is likely that the seamount flows have an important influence on the microbial community structure^35^. The bacteria inhabiting seamounts may have developed special flagellar propellers to adapt to the seamount flows. We previously reported the occurrence of a complex flagella architecture associated with the *Candidatus* Magnetococcus massalia strain MO-1. This bacterium is a chemolithoautotroph, and in chemically stratified sediments, should be able to use reduced sulfur compounds as electron donors in deep sediment layers and oxygen as an electron acceptor in the surface layers^38^. Strain MO-1 produces 12 types of flagellin that are heavily and heterogeneously glycosylated^39^, and they are assembled into 7 flagellar filaments arranged in an intertwined hexagonal array in a sheath^40^. These complex arrangements of flagella have been observed only in MTB from marine coastal sediments and are considered to be an adaptation to life in these habitats. In this study, we observed an even more complicated flagellar architecture that consisted of 19 flagella filaments arranged in a 3:4:5:4:3 array. This structure has not been reported previously, and it probably evolved as an adaptation to life in seamount ecosystems that have strong current flows.

Compared with other oceanic ecosystems, very limited information is available concerning seamount habitats. Here, we report the first systematic analysis of the bacterial community structure and the occurrence of MTB in this unique ecosystem. We found live MTB at all 14 seamount stations and identified 16 novel MTB species, which suggests the occurrence of microbial endemism specific to the Kexue seamount. Interestingly, the station on the steep southeast slope, facing the Mariana-Yap Trench, is rich in minerals (Fig. 2) and has different community composition (Fig. 3) compared to other stations. The geographic properties of the seamount seem to be important in shaping the population structure of the distinctive bacterial communities. Based on this observation we anticipate that the microbial community structure and the MTB species inhabiting other seamounts might be significantly different from those we found at the Kexue seamount located at the junction of the Yap and Mariana trenches. We will undertake studies to gain more knowledge of the microbial communities, biogeography, functions, and evolution of MTB in seamount ecosystems.

## Methods

### Sample collection

During cruise 1602 of the Chinese research vessel *Kexue* (Chinese meaning: science) in the Caroline seamount area from 25 February to 2 April 2016, the ROV *Faxian* (Fugro Geosolutions, Shenzhen, China) undertook 14 dives and collected sediments from various depths (238–2, 023 m) and sampling stations on the seamount (Fig. 1a, Supplementary Fig. S5). The sediments at stations M2-2-2, M2-5, M2-4, M2-3 and M2-22 were collected using a plastic pushcorer (28.3 cm^2^× 35 cm), while a custom-made, steel alloy sediment collector (28.3 cm^2^× 44 cm; Supplementary Fig. S5) was used for collections at the other stations. We also collected mixed sediments from an extensive area of the seamount using a siphon pump-driven suction sampler (7.5 cm diameter, 310 L min^−1^, 84 psi), which was used for collecting both benthos and sediments during two ROV dives.

Approximately 10 g of sampled sediments from each station were transferred to sterile sealed bags and immediately stored at −20 °C for later analyses on both the microbial communities and the physical and chemical characteristics of the sediments. In parallel, sediment for MTB-enrichment was collected from each station and aliquoted into 500-ml plastic bottles that were half-filled with seawater collected above the sediment at that station. Enrichment and collection of MTB involved placing a south-pole magnetic disk (approximately 300 Gauss) on one side of each bottle containing the sediment sample, and a north-pole magnet disk on the opposite side. After 40 min, water samples containing bacteria were drawn from the bottle, adjacent to each magnet, using Pasteur pipettes and were transferred into microtubes^41^.

### DNA extraction, 16S rRNA gene sequencing, and dataset analysis

For the study of microbial communities, total genomic DNA was extracted directly from 0.5-g wet-weight sediment using the FastDNA Spin Kit for Soil (MP Biomedicals, LLC, USA)^13^. The hypervariable region V3-V5 of the bacterial 16S rRNA gene was amplified by PCR using the barcoded universal primers 338 F (5′-ACTCCTACGGGAGGCAGCA-3′) and 806 R (5′-GGACTACHVGGGTWTCTAAT-3′)^42^. Pair-end libraries were constructed using the TruSeq Nano DNA Library Prep Kit (Illumina, San Diego, CA, USA), and high-throughput sequencing was performed using an Illumina MiSeq. 2500 sequencer with the MiSeq Reagent Kit v3 (Illumina), following the manufacturer’s protocols. Raw reads with lower quality scores were filtered using Trimmomatic^43^, as previously described^44^, and pair-end reads were merged using FLASH^45^. On the QIIME platform^46^, OTUs were clustered using UPARSE with a 97% similarity cutoff^47^, and chimeric sequences were identified and removed using UCHIME^48^. The taxonomic classification of each OTU was determined by alignment with the SILVA database (version 123) and by using the RDP classifier tool (70% credibility)^49,50^. The sequences of 21 OTUs that were related to MTB were deposited in the NCBI under accession numbers MF073286-MF073306 (~450 bp).

To study MTB diversity, the enriched MTB were purified using either a ‘capillary racetrack’ method^41^, modified through the use of a 1 ml micropipette tip connected to a 0.2 ml microtube placed in a uniform 300 Gs magnetic field (tube-tip racetrack), or magnetic micromanipulation^51,52^. We used a REPLI-g® Single Cell Kit (QIAGEN, Hilden, Germany) for whole genome amplification (WGA) of the MTB. The amplified genomic DNA was used as a template to amplify the 16S rRNA gene using the bacteria-specific primers 27 F and 1492 R (Sangon Biotech, Shanghai, China). The amplified 16S rRNA genes were purified using the TaKaRa MiniBEST Agarose Gel DNA Extraction Kit Ver.4.0, cloned into pMD®18-T vector (TaKaRa, Japan), transformed into competent *Escherichia coli* Top10 cells, and sequenced with Shanghai Sunny Biotech and TSINGKE Biological Technology (Qingdao, China).

All 16S rRNA gene sequences (~1,470 bp) were aligned using CLUSTAL W multiple alignment, and sequence identities were calculated using the BioEdit sequence alignment editor (version 7.0). We used 97% sequence identity as the OTU threshold. Representative sequences of each OTU were analysed using the BLAST search programme (http://www.ncbi.nlm.nih.gov/BLAST/). Phylogenetic trees were constructed based on neighbor-joining analysis, using the software MEGA 6.0. Bootstrap values were calculated using 1,000 replicates. The 16S rRNA gene sequences were deposited in the NCBI under accession numbers MF099873-MF099891.

### Light microscopy and transmission electron microscopy

A volume of 30 μl from each magnet-enriched and racetrack-collected sample was used to prepare a hanging drop within an applied magnetic field, as previously described^53^. After several minutes, the MTB that accumulated at the north and south poles were counted using a microscope. The polarity of the magnetic field was then reversed to confirm the presumed magnetotactic response of the bacteria, by observing if they swam away from the water droplet edge towards the opposite side of the droplet, in accordance with the reversed polarity. For later analysis, the swimming behaviour was recorded using a camera. The collected MTB were loaded onto carbon-coated copper grids for TEM analysis.

An Olympus CX31 microscope (Olympus, Tokyo, Japan) was used for on board observations. The squash slide technique was used to measure MTB motility with an Olympus BX51 fluorescence microscope in dark-field mode. TEM observations were made using Hitachi HT770 (Japan) and Jeol JEM2100 (Japan) transmission electron microscopes, operating at 100 kV and 200 kV accelerating voltages, respectively. The JEM2100 microscope was equipped with a scanning TEM (STEM) device, which facilitated Z-contrast imaging in high angle annular dark field (HAADF) mode, and an X-MaxN TSR silicon drift detector (Oxford Instruments, UK), which enabled chemical microanalysis by EDXS in the TEM mode.

### Chemical and physical analyses of sediments

Sediments from each station were dried at 80 °C for 3 h. Dry sediment (0.1 g) was used to measure the content of major (Al_2_O_3_, Fe_2_O_3_, MnO, and P_2_O_5_) and trace (Co, Ni, Se, Zn, Pb, and Cu) elements, using inductively coupled plasma-atomic emission spectrometry (ICP-AES; PerkinElmer Optima 3000) and inductively coupled plasma mass spectrometry (ICP-MS; SCIEX Elan 5000, USA), respectively, following nitrolysis^54^.

The granule composition of the Kexue seamount sediments was analysed with approximately 5 g of well-dispersed dry sediment, using an Olympus SZX16 research stereomicroscope equipped with a DP25 digital camera.

## Electronic supplementary material

Supplementary Information
