# Supplementary material for: Bacterial community structure and novel species of magnetotactic bacteria in sediments from a seamount in the Mariana volcanic arc
Source: Sci Rep. 2017 Dec 21;7:17964. doi: 10.1038/s41598-017-17445-4 (PMC5740136; doi:10.1038/s41598-017-17445-4)
Supplement: Supplementary file 1 — Supplementary Information [file 41598_2017_17445_MOESM1_ESM.pdf]

Supplementary Information

Bacterial community structure and novel species of magnetotactic bacteria in sediments from a seamount in the Mariana volcanic arc

Jia Liu[1,2,3#], Wen-Yan Zhang[1,2,5#], Xuegong Li[4,5], Xuegang Li[1,2], Xumiao Chen[6], Jin-Hua Li[5,7], Zhaojie Teng[1,2,3], Cong Xu[1,2,3], Claire-Lise Santini[5,8], Li Zhao[1,2], Yuan Zhao[1,2], Heng Zhang[7], Wei-Jia Zhang[4,5], Kuidong Xu[6], Chaolun Li[1,2], Yongxin Pan[5,7], Tian Xiao[1,2,5]* , Hong-Miao Pan[1,2,5]* and Long-Fei Wu[5,8]*

[1] CAS Key Laboratory of Marine Ecology and Environmental Sciences, Institute of Oceanology, Chinese Academy of Sciences, Qingdao 266071, China.

[2] Laboratory for Marine Ecology and Environmental Science, Qingdao National Laboratory for Marine Science and Technology, Qingdao 266071, China.

[3] University of Chinese Academy of Sciences, Beijing 100864, China.

[4] Laboratory of Deep Sea Microbial Cell Biology, Institute of Deep Sea Science and Engineering, Chinese Academy of Sciences, Sanya, 572000, China.

[5] International Associated Laboratory of Evolution and Development of Magnetotactic Multicellular Organisms (LIA-MagMC), CNRS-CAS, Marseille-Beijing-Qingdao-Sanya.

[6] Department of Marine Organism Taxonomy and Phylogeny, Institute of Oceanology, Chinese Academy of Sciences, Qingdao 266071, China.

[7] Paleomagnetism and Geochronology Lab, Key Laboratory of the Earth's Deep Interior, Institute of Geology and Geophysics, Chinese Academy of Sciences, Beijing 100029, China.

[8] Aix Marseille University, CNRS, LCB, Marseille, France.

[#]These authors contributed equally to this work.

*Correspondence: 1. Long-Fei Wu. LCB, UMR7283, IMM, CNRS; 31, chemin Joseph Aiguier, 13402, Marseille, cedex 20, France; E-mail: wu@imm.cnrs.fr; Tel.: 33-4-9116 4157; Fax: 33-4-9171 8914.

2. Tian Xiao, KLMEES, IOCAS; 7, Nanhai Road, 266071, Qingdao, Shandong, P. R. China; E-mail: txiao@qdio.ac.cn; Tel.: 86-532-82898584; Fax: 86-532-82898586.

3. Hongmiao Pan, KLMEES, IOCAS; 7, Nanhai Road, 266071, Qingdao, Shandong, P. R. China; E-mail: panhongmiao@qdio.ac.cn; Tel.: 86-532-82898593; Fax: 86-532-82898586.

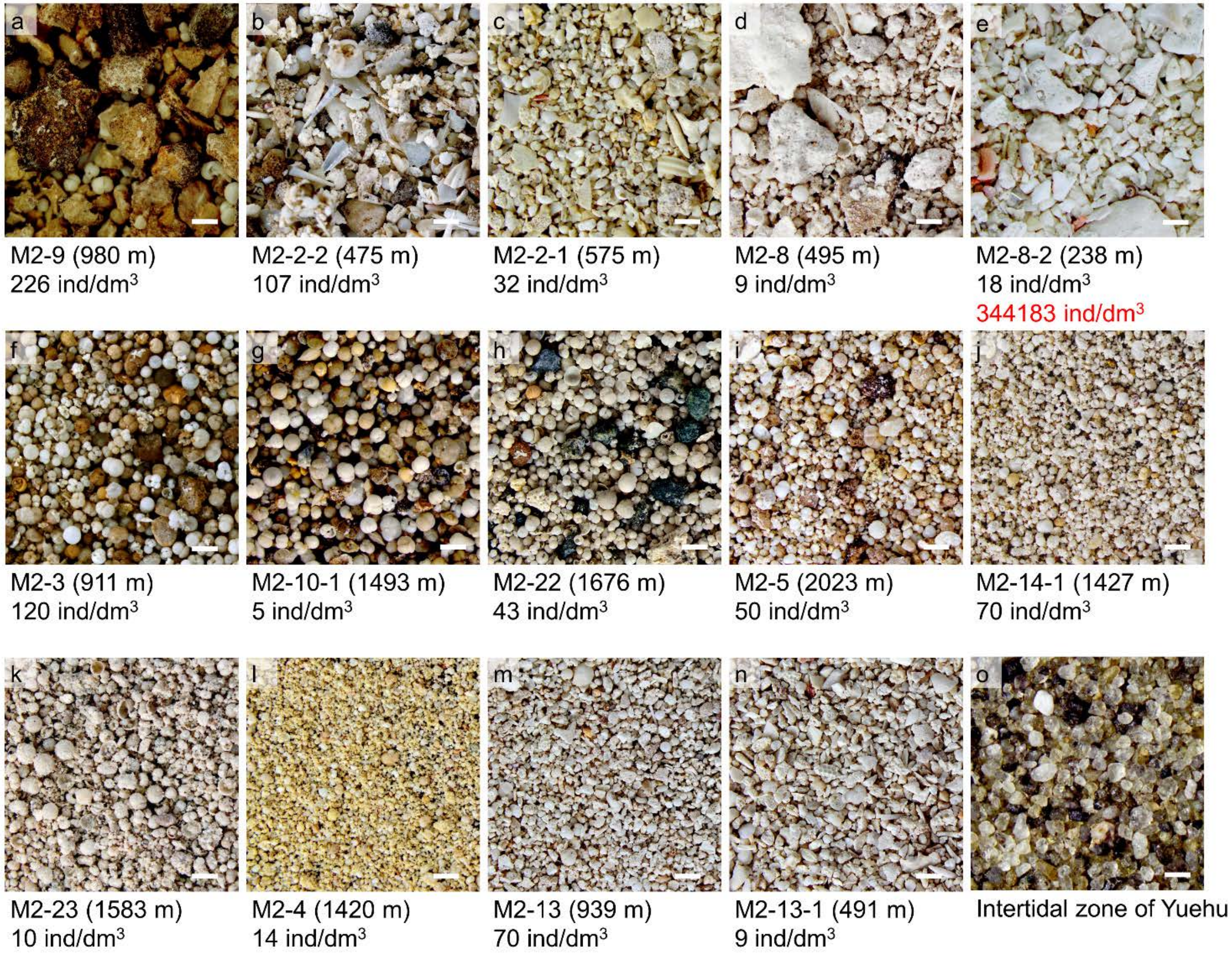

Supplementary Figure S1. Stereomicroscope images of sediments.

Panels a–n show stereomicroscope images of the sediments from 14 stations of the Kexue seamount. Red numbers indicate MTB abundance, determined after laboratory storage. Panel o shows sediments from the intertidal zone at Yuehu. The scale bars are 1 mm.

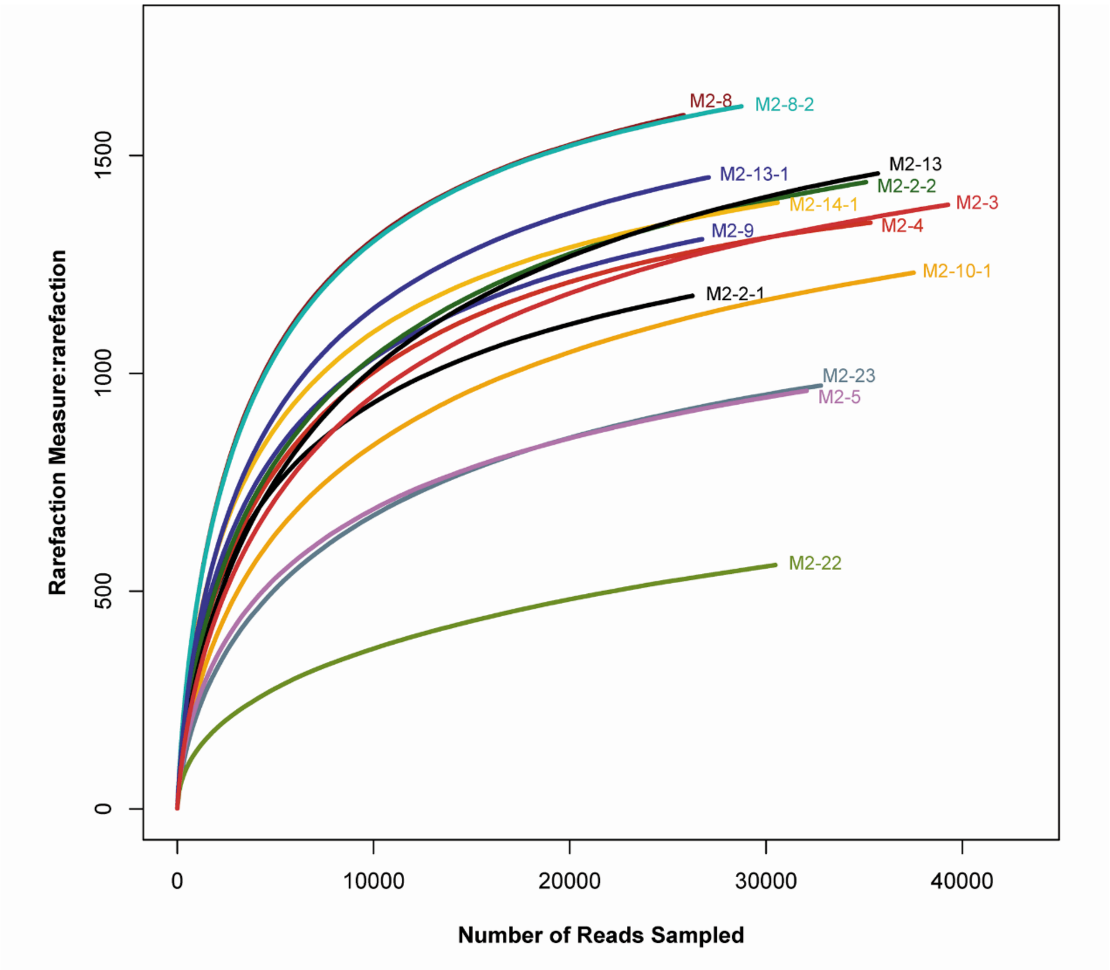

Supplementary Figure S2. Rarefaction curves at 3% difference levels.

Rarefaction curves for seamount sediment bacterial communities using 16S rRNA gene sequences at 3% difference levels.

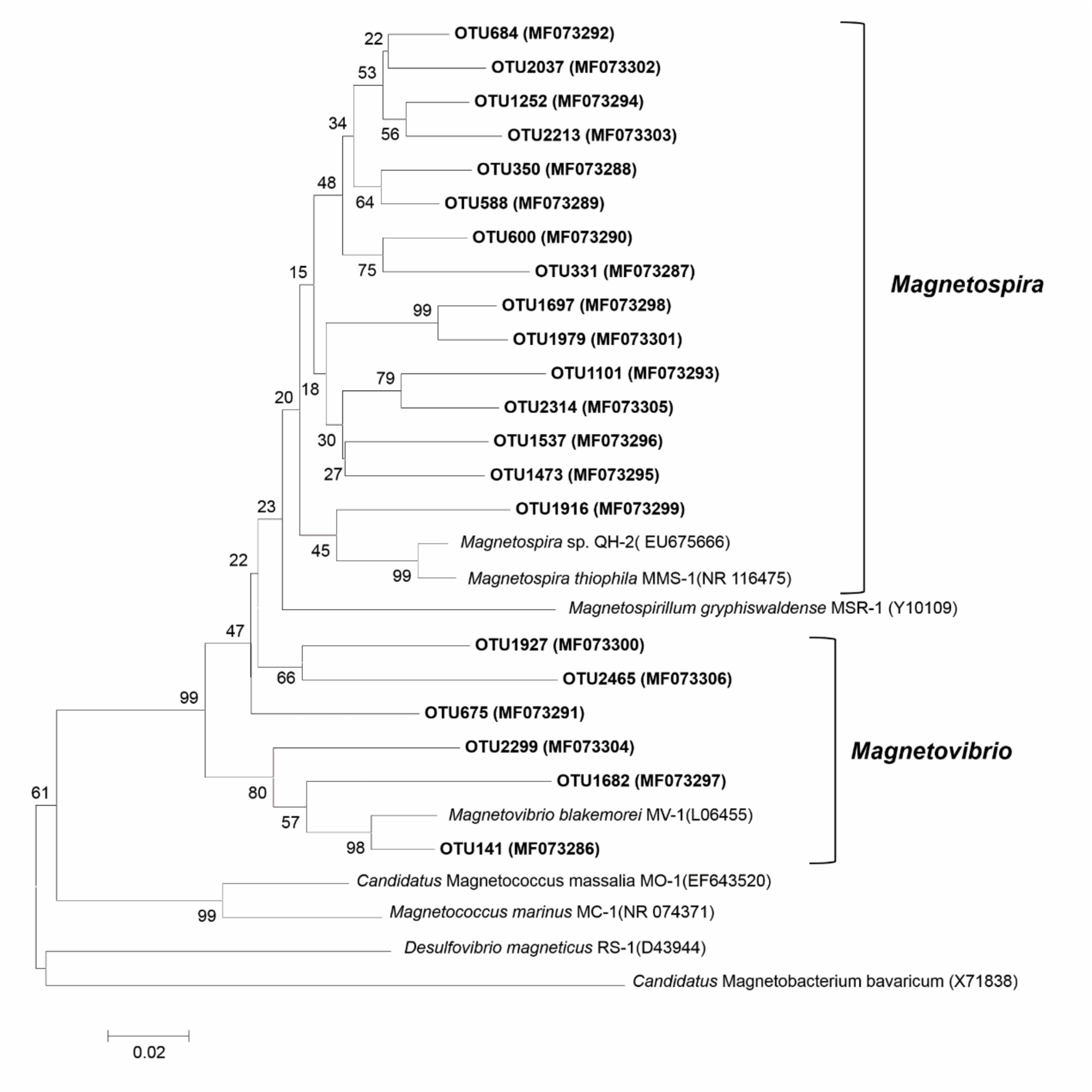

Supplementary Figure S3. Phylogenetic tree based on partial 16S rRNA gene sequences (approximately 450 bp) of the 21 OTUs related to magnetotactic bacteria from Kexue seamount.

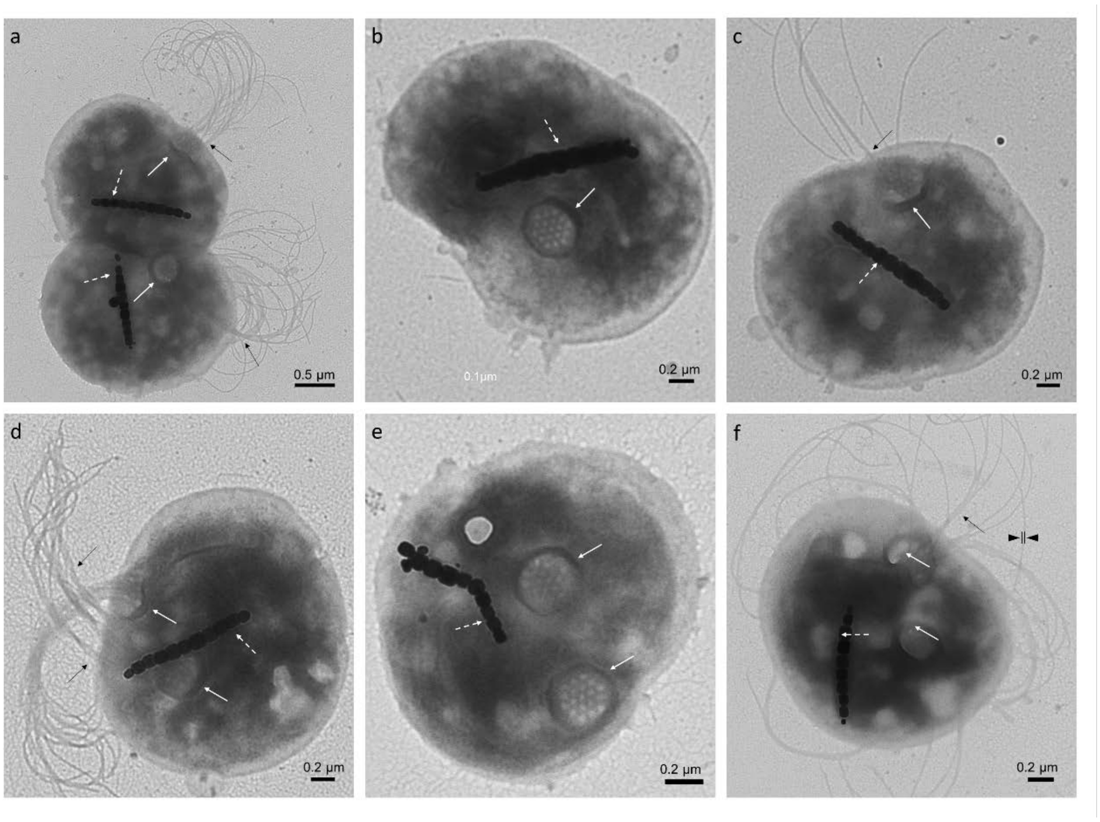

Supplementary Figure S4. Flagella and Flagellar base platform.

Flagella, flagellar base platforms, and magnetosomes are indicated by black arrows, white arrows, and white dashed arrows, respectively. Panels a–c show magnetotactic cocci each having one flagellar base platform. Panels d–f show magnetotactic cocci each having two flagellar base platforms. The diameter of the flagella is indicated by black arrow heads.

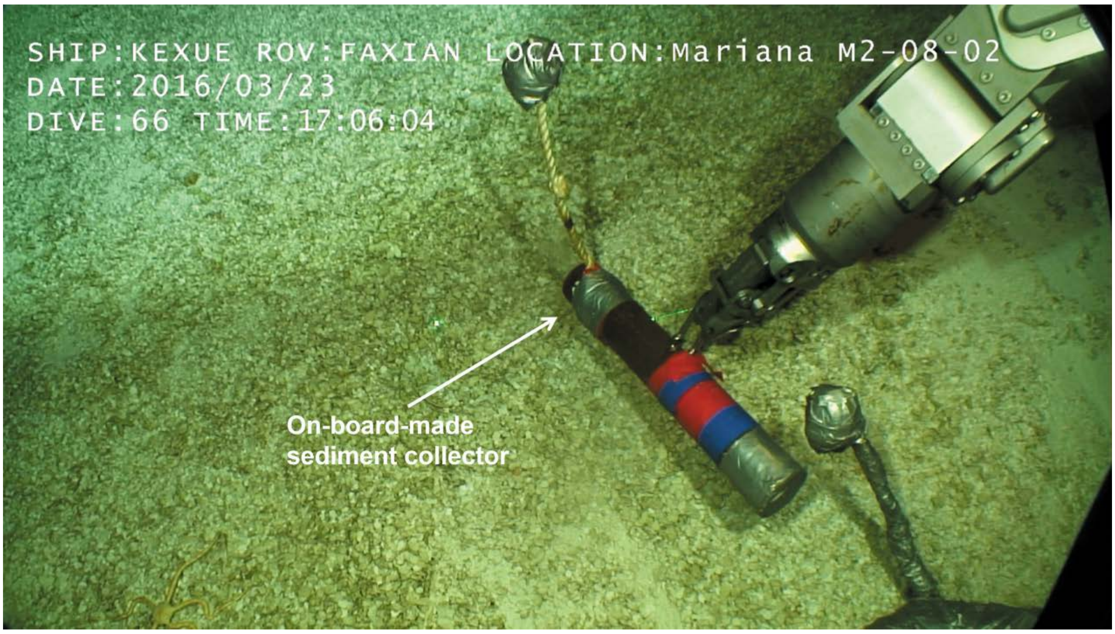

Supplementary Figure S5. ROV sampling and custom-made sediment collector.

Station	Reads	OTUs	Ace	Chao	Coverage	Shannon	Simpson
M2-2-1	26,252	1,178	1,344	1,374	0.991	5.65	0.0087
M2-8-2	28,749	1,613	1,739	1,779	0.992	6.35	0.0039
M2-23	32,791	972	1,153	1,171	0.993	4.00	0.0968
M2-22	30,474	560	934	790	0.994	3.93	0.0462
M2-10-1	37,520	1,231	1,445	1,474	0.993	4.86	0.0280
M2-5	32,069	960	1,147	1,174	0.993	4.87	0.0214
M2-13	35,695	1,459	1,655	1,666	0.992	5.22	0.0204
M2-13-1	27,072	1,450	1,607	1,600	0.991	5.78	0.0136
M2-3	39,274	1,387	1,568	1,620	0.993	5.02	0.0256
M2-9	26,739	1,308	1,464	1,475	0.991	5.66	0.0123
M2-4	35,320	1,346	1,462	1,464	0.994	5.24	0.0297
M2-2-2	35,092	1,439	1,607	1,612	0.993	5.32	0.0253
M2-14-1	30,589	1,392	1,530	1,572	0.993	5.95	0.0078
M2-8	25,797	1,593	1,740	1,787	0.990	6.31	0.0046

Supplementary Table S1. Richness and diversity of bacteria at 3% difference levels.

Chao and ACE are the estimated bacterial richness values. Shannon and Simpson refer to the calculated bacterial diversity values. Coverage is the Good’s coverage.

		Shannon	MTB OTUs	MTB reads	Al	Fe	Mn	Co	Cu	Zn	Se	Pb	Ni
MTB OTUs	P	0.809**											
	Sig.	<0.001											
MTB_reads	P	0.610*	0.797**										
	Sig.	0.020	0.001										
Al	P	-0.705**	-0.510	-0.334									
	Sig.	0.005	0.063	0.243									
Fe	P	-0.804**	-0.609*	-0.439	0.892**								
	Sig.	0.001	0.021	0.117	<0.001								
Mn	P	-0.645*	-0.540*	-0.396	0.778**	0.725**							
	Sig.	0.013	0.046	0.161	0.001	0.003							
Co	P	-0.428	-0.410	-0.402	0.307	0.572*	0.440						
	Sig.	0.127	0.145	0.154	0.285	0.033	0.116						
Cu	P	-0.734**	-0.545*	-0.422	0.686**	0.912**	0.592*	0.534*					
	Sig.	0.003	0.044	0.133	0.007	<0.001	0.026	0.049					
Zn	P	-0.830**	-0.525	-0.367	0.834**	0.917**	0.774**	0.395	0.904**				
	Sig.	<0.001	0.054	0.196	<0.001	<0.001	0.001	0.162	<0.001				
Se	P	0.031	-0.024	0.054	0.241	0.125	0.293	0.061	-0.013	0.081			
	Sig.	0.916	0.935	0.855	0.407	0.670	0.309	0.836	0.964	0.782			
Pb	P	-0.626*	-0.367	-0.362	0.671**	0.769**	0.638*	0.717**	0.616*	0.697**	0.207		
	Sig.	0.017	0.197	0.203	0.009	0.001	0.014	0.004	0.019	0.006	0.477		
Ni	P	-0.779**	-0.659*	-0.539*	0.595*	0.680**	0.841**	0.52	0.570*	0.716**	0.066	0.698**	
	Sig.	0.001	0.010	0.047	0.025	0.008	<0.001	0.057	0.033	0.004	0.823	0.006	
IP	P	0.046	0.011	0.072	0.407	0.282	0.567*	0.130	0.216	0.330	0.160	0.228	0.253
	Sig.	0.875	0.971	0.807	0.148	0.328	0.035	0.659	0.458	0.249	0.585	0.433	0.383

Supplementary Table S2. Correlation analysis between bacterial diversity and chemical elements in sediments at Kexue seamount.

*: Correlation is significant at the 0.05 level (two-tailed); **: Correlation is significant at the 0.01 level (two-tailed); P: Pearson correlation coefficient; Sig. : Significance, P value; N=14; IP indicates the concentration of inorganic phosphorus (μg/g).

OTU	MTB related Blast-hit results	Identity	Novel species
OTU6	Magnetic coccus rj516 from Itaipu lagoon (Y13209)	97.3%	known species
OTU12	Magnetic coccus rj12 from Itaipu lagoon (Y13215)	97.3%	known species
OTU1	Magnetic coccus rj516 from Itaipu lagoon (Y13209)	98.2%	known species
OTU3	Magnetic coccus rj12 from Itaipu lagoon (Y13215)	96.7%	novel species*
OTU8	Magnetic coccus rj12 from Itaipu lagoon (Y13215)	96.2%	novel species*
OTU13	Magnetic coccus MRG-138 from HuiQuan Bay (KM099217)	93.0%	novel genus[1]
OTU19	Magnetic coccus MRG-138 from HuiQuan Bay (KM099217)	92.7%	novel genus[1]
OTU14	Magnetic coccus rj12 from Itaipu lagoon (Y13215)	93.0%	novel genus[2]
OTU17	Magnetic coccus rj12 from Itaipu lagoon (Y13215)	92.6%	novel genus[2]
OTU2	Magnetic coccus MRG-138 from HuiQuan Bay (KM099217)	90.9%	novel genus
OTU4	Magnetic coccus rj12 from Itaipu lagoon (Y13215)	94.3%	novel genus
OTU5	Magnetic coccus M-67 from Jiaozhou Bay (EF371491)	93.2%	novel genus
OTU7	Magnetic coccus MRG-138 from HuiQuan Bay (KM099217)	89.8%	novel genus
OTU9	Magnetic coccus rj12 from Itaipu lagoon (Y13215)	94.7%	novel genus
OTU10	Magnetic coccus M-67 from Jiaozhou Bay (EF371491)	88.6%	novel genus
OTU11	Magnetic coccus M-67 from Jiaozhou Bay (EF371491)	93.2%	novel genus
OTU15	Magnetic coccus MRC-12 from Yuehu lagoon (KJ591590)	89.3%	novel genus
OTU16	Magnetic coccus M-67 from Jiaozhou Bay (EF371491)	90.2%	novel genus
OTU18	Magnetic coccus rj12 from Itaipu lagoon (Y13215)	91.9%	novel genus

Supplementary Table S3. MTB-related OTUs identified from the clone library.

*OTU3 and OTU8 are novel species belonging to a known genus. [1]OTU13 and OTU19 belong to a novel genus. [2]OTU14 and OTU17 belong to a novel genus. OTU1, OTU6 and OTU12 are known species. Other OTUs represented 10 novel genera, respectively.
